# *RBM20*-Associated Ventricular Arrhythmias in a Patient with Structurally Normal Heart

**DOI:** 10.3390/genes12010094

**Published:** 2021-01-13

**Authors:** Yuriy Vakhrushev, Alexandra Kozyreva, Andrey Semenov, Polina Sokolnikova, Tamara Lubimtseva, Dmitry Lebedev, Natalia Smolina, Sergey Zhuk, Lubov Mitrofanova, Elena Vasichkina, Anna Kostareva

**Affiliations:** 1 Almazov National Medical Research Centre, Institute of Molecular biology and Genetics, 197341 Saint Petersburg, Russia; klyushina_aa@almazovcentre.ru (A.K.); Semenov_ap@almazovcentre.ru (A.S.); sokolnikova_ps@almazovcentre.ru (P.S.); lubimtseva_t@almazovcentre.ru (T.L.); Lebedev@almazovcentre.ru (D.L.); bonifacii.vip@gmail.com (N.S.); s.v.zhuk@gmail.com (S.Z.); Mitrofanova_LB@almazovcentre.ru (L.M.); Vasichkina_ES@almazovcentre.ru (E.V.); anna.kostareva@ki.se (A.K.); 2 Department of Women’s and Children’s Health and Center for Molecular Medicine, Karolinska Institute, 17177 Stockholm, Sweden

**Keywords:** *RBM20*, ventricular tachycardia, cardiomyopathy

## Abstract

*RBM20* (RNA-binding motif protein 20) is a splicing factor targeting multiple cardiac genes, and its mutations cause cardiomyopathies. Originally, *RBM20* mutations were discovered to cause the development of dilated cardiomyopathy by erroneous splicing of the gene *TTN* (titin). Titin is a giant protein found in a structure of the sarcomere that functions as a molecular spring and provides a passive stiffness to the cardiomyocyte. Later, *RBM20* mutations were also described in association with arrhythmogenic right ventricular cardiomyopathy and left ventricular noncompaction cardiomyopathy. Here, we present a clinical case of a rare arrhythmogenic phenotype and no structural cardiac abnormalities associated with a *RBM20* genetic variant of uncertain significance.

## 1. Introduction

Dilated cardiomyopathy (DCM) is one of the most common causes of progressive heart failure, heart transplantation and sudden cardiac death (SCD), that has an estimated prevalence 1 in 2500 individuals [1,2]. DCM is inherited in about 30–40% of all cases, and there are more than 40 genes found to be associated with DCM in humans [3,4]. One of them is the *RBM20* gene, mutations of which cause DCM in approximately 2–4.8% of all cases [5]. The first mutation in *RBM20* was identified in 2009 using genome-wide linkage analysis in two large families with autosomal dominant DCM [6].

*RBM20* encodes for a protein consisting of 1227 amino acids and containing several conserved functional domains: a leucine (L)-rich region at the N-terminus, two zinc finger (ZnF) domains (ZnF1 and ZnF2), an RNA recognition motif (RRM), an arginine-serine (RS) domain and a glutamate E-rich region between the RS domain and the ZnF2 domain at the C-terminus [7,8]. The *RBM20* protein (RNA binding motif protein-20) has been identified as a key RNA splicing factor for genes that are selectively expressed in the heart, and has been demonstrated to regulate alternative splicing events of selected genes involved in sarcomere assembly, ion transport and structural organization of cardiomyocytes [8,9,10]. The essential structural domains required for splicing activities are not fully identified, although *RBM20* mutations in the RSRSP (arginine-serine-arginine-serine-proline) stretch, a hotspot of missense mutations found in patients with idiopathic DCM, and the E-rich region, have been demonstrated to affect the regulation of exon splicing [11]. *RBM20* exon targets were initially identified by RNA-seq analyses in an Rbm20-/- rat’s heart and in human heart tissues derived from subjects carrying *RBM20* mutations. Thus a set of 31 genes regulated by *RBM20* have been found [8]. The most studied and well-known target of *RBM20* is a *TTN* gene encoding for a giant protein that provides passive stiffness to the sarcomere. Therefore, a primary molecular mechanism attributed to *RBM20* mutations was suggested as a change in the N2B/N2BA titin isoforms ratio, resulting in excentric myocardial remodelling and DCM development. Later on, genes including *CaMKII*, *CACNA1C*, *LDB3*, *LMO7*, *FHOD3*, *PDLIM3*, *RTN4*, *TRDN*, *OBSCN, RYR2* and many others have been specified as targets of *RBM20* [8,12,13,14,15]. Taking into account the effect of *RBM20* mutations on splicing of the above-mentioned ion-channel encoding genes, *RBM20*-associated DCM is often accompanied by arrhythmic phenotypes such as nonsustained ventricular tachycardia and atrial fibrillation [16]. However, the isolated arrhythmic phenotype has never been reported in patients with *RBM20* variants to our knowledge. Here, we present the first description of a patient with *RBM20*-associated ventricular arrhythmias and a structurally normal heart.

## 2. Materials and Methods

The study was performed according to the Declaration of Helsinki, and approval was obtained from the Almazov National Medical Research Centre Ethical Committee, approval number 2016/54. Written informed consent was obtained from the patient. Routine clinical examination included physical examination, 12-lead electrocardiography and Holter ECG monitoring, transthoracic echocardiography, endomyocardial biopsy, biochemical and hormone tests.

### Target and Whole Exome Sequencing

For genetic testing, DNA was extracted from whole blood with a FlexiGene Kit according to the manufacturer’s recommendations. A targeted panel of 172 cardiomyopathy-associated genes was analyzed using the SureSelect Target Enrichment System (Agilent; Waldbronn, Germany) using an Illumina MiSeq instrument. The list of studied genes is presented in Supplemental Table S1 Data processing and filter strategy were performed as described earlier [17].

## 3. Results

### 3.1. Clinical Presentation

**Figure 1:**
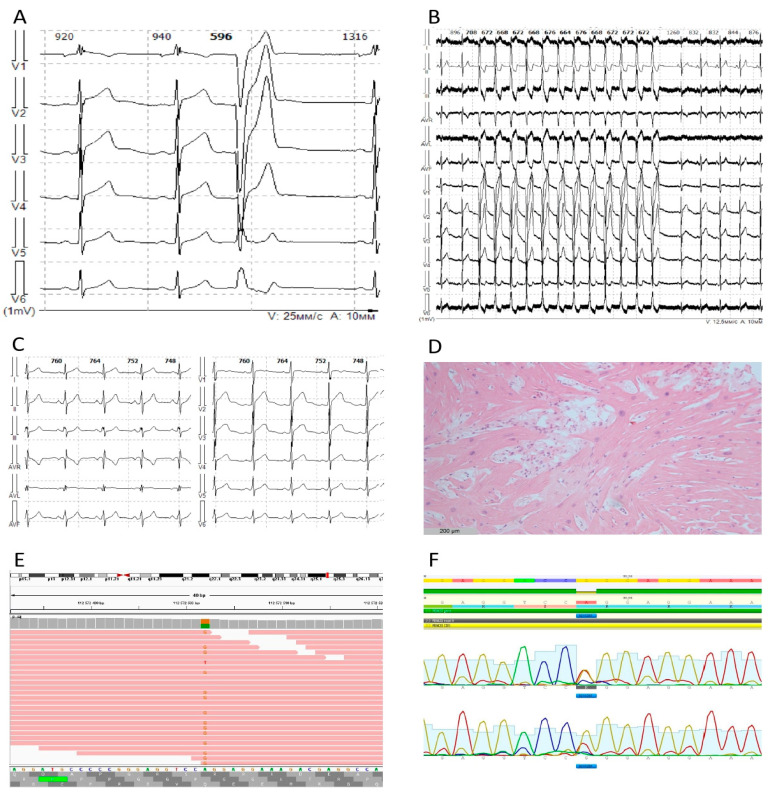
(**A**) Sinus rhythm, single premature ventricular beat. (**B**) Episode of nonsustained ventricular tachycardia. (**C**) ST segment ascending elevation in right precordial leads. (**D**) Myocardial biopsy sample demonstrating cardiomyocyte hypertrophy, disarray, lipomatosis and no gross areas of fibrosis. (**E**) Sequencing reads in IGV (integrative genomics viewer) format, demonstrating c.A2347G:p.R783G substitution in approximately 50% of the reads. (**F**) Sanger sequencing data confirming c.A2347G:p.R783G substitution.

A 26-years old male was referred to the cardiological department due to repeated syncope episodes associated with emotional stress but not physical activity. His family history was unremarkable and no inherited cardiac disorders or sudden cardiac death episodes were reported. Echocardiography documented normal heart morphology, no signs of congenital heart disorders, chamber dilation or ventricular hypertrophy. Holter monitoring revealed frequent polymorphic ventricular extra beats and paroxysmal episodes of nonsustained polymorphic ventricular tachycardia, transient ST non-Brugada-like elevation in V1-V2 precordial leads and partial right bundle-branch block (Figure 1A–C). Novocainamid provocation did not confirm Brugada syndrome, and an invasive electrophysiology test failed to provoke the arrhythmic episodes. Myocardial biopsy demonstrated moderate cardiomyocyte hypertrophy, dystrophy and mild fibrosis; no signs of inflammation or myocarditis were found (4CD45+, 4CD3+/mm^2^, HLA-DR “-”, Figure 1D). Due to the repeated syncope episodes linked to polymorphic ventricular tachycardia, an implantable cardioverter defibrillator (ICD) was implanted as a secondary prevention and the patient was discharged with β-blocker therapy (75 mg of metoprolol). Within the next four years, the palpitation and ventricular tachycardia progressed over time and several episodes of ICD discharges were registered. The antiarrhythmic therapy was escalated to 100 mg of metoprolol and 80 mg of sotalol daily and the patient remained clinically stable within the next years. No myocardial remodelling was noted over time, and echocardiography parameters remained within the normal range until the current age of 32 years.

### 3.2. Sequencing and Genetic Analysis

Genetic analysis using 172 target cardiomyopathy-associated genes panel revealed a variant in *RBM20* ((NM 001134363:c.A2347G:p.R783G, rs1287523613), MAF = 0.000006, 1/156478, GnomAD_exome) which was further confirmed by Sanger sequencing (Figure 1E,F). This variant was previously reported in a patient with DCM (https://www.ncbi.nlm.nih.gov/clinvar/variation/572926/) and is considered to be damaging or pathogenic according to six prediction programs (DNAA, EIGEN PC, FATHMM-MKL, FATHMM-XF, MutationTester, SIFT and SIFT4G), has a 25.3 CADD PHRED prediction score and, according to ACMG criteria, can be interpreted as a variant of uncertain significance.

## 4. Discussion

Here, we present a clinical case of *RBM20*-associated ventricular arrhythmia in a patient with no echocardiographic features of structural heart disease or cardiomyopathy phenotype. The malignant character of the arrhythmic syndrome, i.e., several syncope episodes and sudden cardiac death with no effect of antiarrhythmic drugs in a patient of 31 years of age, initially raised suspicion towards inherited channelopathy such as catecholaminergic polymorphic ventricular tachycardia or Brugada syndrome. However, no pathogenic, likely pathogenic variants, or variants of unknown significance were detected in the genes associated with any of the inherited arrhythmic syndromes. Due to the implanted ICD there was no possibility for MRI assessment. Therefore, arrhythmogenic cardiomyopathy (ACM) criteria were only possible to assess using an echocardiography imaging modality with an RV (right ventricular) dimension of 25.5 mm in a parasternal position, which was not enough to consider ACM. Given the absence of repolarization and depolarization abnormalities corresponding to ACM criteria, and no gross morphological features of fibrosis, along with a high proportion of residual myocytes, the ACM diagnosis was excluded (for detailed criteria analysis see Supplemental Table S2). However, there was still the potential that chamber dilation would take place later on upon disease progression resulting in either DCM or ACM.

The only genetic variant of interest identified in the patient was a variant in the *RBM20* gene. According to the American College of Medical Genetics and Genomics classification, this variant has been classified as a variant of unknown significance. Moreover, earlier, this variant was reported in a patient with DCM. However no further detailed clinical information was available. Taken together, these data allowed us to consider the identified *RBM20* variant as clinically significant in relation to the observed phenotype and to suggest its role in the development of arrhythmogenesis.

The presence of ventricular arrhythmias, despite the gross myocardial fibrosis and chamber remodelling, can potentially be explained by the effect of *RBM20* on splicing of genes encoding for ion channels such as *CACNA1C*, *RYR2* and *SCN5A* [12,18]. This hypothesis is well in line with the data recently reported by Hey and coauthors who demonstrated that DCM patients with *RBM20* variants present a more severe form of disease compared to overall DCM patients [19]. Furthermore, Parikh and coauthors demonstrated that patients with *RBM20* variants more often reveal sustained ventricular arrhythmia and nonsustained ventricular tachycardia compared to the overall DCM population and patients with *TTN*-truncated variants (*TTNtv*). Moreover, according to their data, *RBM20* variants worsen the prognosis of the patients carrying *LMNA* pathogenic variants [16]. Therefore, the leading pathological process resulting in the arrhythmic phenotype in our patient could exist primarily at the molecular level without major histological abnormalities and chamber remodelling.

Of note, the identified genetic variant does not reside in *RBM20* regions enriched for cardiomyopathy-associated variants. These variants generally occur within the following gene-coding regions: c.1601-1640 (RNA-recognition motif (RRM)-type RNA-binding domain), c.1881-1920 (arginine/serine (RS)-rich region domain) and c.2721-2760 (exon 11, glutamate-rich region). Patients with genetic variants found in these regions are more likely to have ventricular arrhythmia, shorter PR intervals and family history of SCD compared to the patients with variants outside of these windows [16].

This underlines the potential existence of the new not-yet identified gene areas linked to the arrhythmogenic phenotype and underscores the importance of the detailed electrophysiological and RNA expression studies using cardiomyocyte cellular models including induced pluripotent stem cells-derived cardiomyocytes.

A major limitation of the current study is the unavailability of more family members for genotyping, which made it impossible to make a deep genotype-phenotype analysis and to draw a strong conclusion regarding the variant identified. Further data collection and reporting regarding phenotypic manifestation of *RBM20* variants will allow accumulatiom of additional knowledge of *RBM20*-associated cardiomyopathy phenotypes. Deeper morphological, cellular or expression analyses, which were not performed in the current study, will additionally address the questions regarding this form of cardiomyopathy.

## 5. Conclusions

In summary, we describe a patient with a ventricular arrhythmic phenotype and need of ICD implantation despite a structurally normal heart associated with an *RBM20* genetic variant. The described clinical case broadens the spectrum of genetic causes of inherited arrhythmic disorders and underscores the complex aetiology of heart rhythm abnormalities in cardiomyopathies associated with an unfavourable genetic cause such as *LMNA* and *RBM20*-associated cases.

## Supplementary Materials

The following are available online at https://www.mdpi.com/2073-4425/12/1/94/s1, Table S1: List of studied genes. (Almazov_Comprehensive_Cardiac_Panel) 172 Target IDs resolved to 172 targets comprising 3271 regions. Table S2: ARVC criteria of patient.
