# Supplementary material for: RBM20-Associated Ventricular Arrhythmias in a Patient with Structurally Normal Heart
Source: Genes (Basel). 2021 Jan 13;12(1):94. doi: 10.3390/genes12010094 (PMC7828370; doi:10.3390/genes12010094)
Supplement: Supplementary file 1 [file genes-12-00094-s001.pdf]

Supplementary Table S1

List of studied genes. (Almazov_Comprehensive_Cardiac_Panel)

172 Target IDs resolved to 172 targets comprising 3271 regions.

	Gene name	Interval	Regions	Size
1.	ABCC9	chr12:21801034-21936684	41	5759
2.	ACADVL	chr17:7217136-7225107	20	2735
3.	ACTA1	chr1:229431489-229433125	6	1254
4.	ACTC1	chr15:34790402-34794818	6	1254
5.	ACTN2	chr1:236686664-236762629	23	3342
6.	ACVR2B	chr3:38454313-38483342	11	1759
7.	AGK	chr7:141555457-141652934	17	1670
8.	AKAP9	chr7:91941090-92110169	50	13065
9.	ALPK3	chr15:84816837-84868466	14	6004
10.	ANK2	chr4:112904484-113381650	56	13912
11.	ANKRD1	chr10:90912856-90921037	9	1140
12.	ANO5	chr11:22193483-22279775	22	3182
13.	BAG3	chr10:119651666-119677292	5	1834
14.	BRAF	chr7:140726484-140924713	21	2799
15.	CACNA1C	chr12:1971053-2691209	56	8643
16.	CACNA2D1	chr7:81950382-82443469	41	4204
17.	CACNB2	chr10:18140727-18539734	20	2669
18.	CALM1	chr14:90397221-90404727	7	596
19.	CALM2	chr2:47160766-47176551	8	929
20.	CALM3	chr19:46601425-46609163	7	593
21.	CALR3	chr19:16479121-16496139	9	1335
22.	CASQ2	chr1:115701231-115768551	11	1420
23.	CAV3	chr3:8733867-8745877	2	496
24.	CBL	chr11:119206408-119308087	18	3163
25.	CDH2	chr18:27952143-28177032	18	3179

26.	CMYA5	chr5:79689898-79799626	13	12470
27.	CRELD1	chr3:9934429-9944589	11	1697
28.	CRYAB	chr11:111908754-111911734	4	867
29.	CSRP3	chr11:19182660-19192458	5	685
30.	CTNNA3	chr10:65920320-67648780	19	3212
31.	DES	chr2:219418453-219426000	9	1593
32.	DMD	chrX:31121823-33339275	87	13178
33.	DMPK	chr19:45770471-45782381	16	2715
34.	DNAAF1	chr16:84145431-84178416	15	2639
35.	DNAAF3	chr19:55159052-55166658	12	2146
36.	DPP6	chr7:153887674-154892490	29	3721
37.	DSC2	chr18:31068005-31101981	17	3082
38.	DSG2	chr18:31498242-31546753	15	3666
39.	DSP	chr6:7541906-7585888	24	9096
40.	DTNA	chr18:34755967-34890450	26	3138
41.	DYSF	chr2:71453989-71686502	58	7650
42.	EMD	chrX:154379475-154381207	6	885
43.	EYA4	chr6:133274771-133531236	21	2477
44.	FHL1	chrX:136196793-136210035	8	1312
45.	FHL2	chr2:105361273-105399555	10	1866
46.	FHOD3	chr18:36297826-36779540	29	5450
47.	FKRP	chr19:46755441-46756948	1	1508
48.	FKTN	chr9:105575023-105640133	13	1926
49.	FLNA	chrX:154348839-154371255	47	8901
50.	FLNC	chr7:128830628-128858533	48	9138
51.	FXN	chr9:69035773-69099944	6	795
52.	GAA	chr17:80104577-80119341	20	3264
53.	GATA4	chr8:11708303-11758485	6	1452
54.	GATA5	chr20:62464826-62475531	6	1314

55.	GATA6	chr18:22171135-22200833	6	1908
56.	GATAD1	chr7:92447720-92456572	5	910
57.	GDF1	chr19:18868587-18870317	2	1159
58.	GJA5	chr1:147758152-147759248	1	1097
59.	GLA	chrX:101397799-101407913	8	1480
60.	GPD1L	chr3:32106702-32165920	8	1216
61.	HAND1	chr5:154475796-154478018	2	688
62.	HCN4	chr15:73322471-73368280	8	3772
63.	HFE	chr6:26087431-26094421	8	1248
64.	HRAS	chr11:532626-534332	5	752
65.	ILK	chr11:6604262-6610621	12	1714
66.	ISPD	chr7:16091685-16421332	10	1556
67.	JPH2	chr20:44114786-44186715	6	2222
68.	JUP	chr17:41755734-41771864	13	2498
69.	KCNA5	chr12:5044138-5045999	1	1862
70.	KCND3	chr1:111776067-111982736	7	2108
71.	KCNE1	chr21:34449235-34449644	1	410
72.	KCNE2	chr21:34370469-34370860	1	392
73.	KCNE3	chr11:74457242-74457573	1	332
74.	KCNE5	chrX:109624582-109625030	1	449
75.	KCNH2	chr7:150945355-150977923	16	4177
76.	KCNJ2	chr17:70175030-70176333	1	1304
77.	KCNJ5	chr11:128911264-128916741	2	1300
78.	KCNJ8	chr12:21765713-21773626	2	1315
79.	KCNQ1	chr11:2444676-2848013	20	2635
80.	KRAS	chr12:25209785-25245394	6	828
81.	LAMA4	chr6:112109427-112254160	40	6637
82.	LAMP2	chrX:120428474-120469179	11	1736
83.	LDB3	chr10:86668682-86732986	16	2840

84.	LEFTY2	chr1:225937411-225941150	4	1201
85.	LMNA	chr1:156114909-156139849	17	2729
86.	LMOD3	chr3:69109085-69122396	3	1743
87.	LRRC10	chr12:69609995-69610848	1	854
88.	LZTR1	chr22:20982362-20997358	22	3249
89.	MAP2K1	chr15:66387338-66490625	12	1462
90.	MAP2K2	chr19:4090588-4123885	11	1423
91.	MIB1	chr18:21741574-21864676	21	3441
92.	MMP21	chr10:125766652-125775831	7	1850
93.	MRAS	chr3:138372874-138402279	5	727
94.	MYBPC3	chr11:47331861-47352657	34	4515
95.	MYBPHL	chr1:109294229-109307001	8	1225
96.	MYH6	chr14:23382030-23407233	37	6560
97.	MYH7	chr14:23412844-23433742	38	6568
98.	MYL2	chr12:110911067-110920539	7	675
99.	MYL3	chr3:46858234-46863400	6	708
100.	MYL4	chr17:47209413-47223052	6	807
101.	MYLK2	chr20:31819571-31833807	12	2031
102.	MYOF	chr10:93306953-93482204	55	7397
103.	MYOM1	chr18:3067252-3215233	37	5799
104.	MYOT	chr5:137870642-137887395	9	1677
105.	MYOZ2	chr4:119136516-119186210	5	895
106.	MYPN	chr10:68106715-68210465	21	4521
107.	NEBL	chr10:20785737-21173843	34	4296
108.	NEXN	chr1:77916097-77942923	13	2302
109.	NF1	chr17:31095198-31378941	63	10271
110.	NKX2-5	chr5:173232559-173235093	5	1295
111.	NKX2-6	chr8:23702441-23706608	2	946
112.	NPPA	chr1:11845993-11847694	3	522

113.	NRAS	chr1:114708525-114716170	4	650
114.	NUP155	chr5:37291890-37370987	35	4884
115.	PDLIM3	chr4:185502284-185535444	9	1463
116.	PKD1L1	chr7:47775133-47948450	57	9726
117.	PKP2	chr12:32792414-32896741	15	2963
118.	PLEC	chr8:143916167-143975379	43	15845
119.	PLEKHM2	chr1:15684549-15733944	20	3460
120.	PLN	chr6:118558912-118559090	1	179
121.	PPA2	chr4:105369715-105474060	14	1350
122.	PPP1CB	chr2:28752115-28799313	9	1363
123.	PRDM16	chr1:3069250-3433821	18	4215
124.	PRKAG2	chr7:151557191-151876630	19	2353
125.	PSEN1	chr14:73148010-73219299	12	1913
126.	PSEN2	chr1:226880636-226895589	11	1666
127.	PTPN11	chr12:112419102-112504774	16	2163
128.	RAF1	chr3:12584504-12618731	17	2347
129.	RANGRF	chr17:8288779-8289946	4	733
130.	RBM20	chr10:110644445-110835988	14	3964
131.	RIT1	chr1:155900378-155910895	7	859
132.	RRAS	chr19:49635566-49640108	6	777
133.	RYR2	chr1:237042512-237832657	105	17087
134.	SALL4	chr20:51784255-51802418	4	3242
135.	SCN10A	chr3:38697339-38794020	27	6411
136.	SCN1B	chr19:35030811-35039711	5	1116
137.	SCN2B	chr11:118166877-118176441	4	728
138.	SCN3B	chr11:123634133-123653811	5	748
139.	SCN4B	chr11:118137017-118152683	5	787
140.	SCN5A	chr3:38550311-38633317	29	6785
141.	SCNN1G	chr16:23186262-23215479	12	2190

142.	SDHA	chr5:218346-256430	16	2449
143.	SGCD	chr5:156329567-156759400	9	1182
144.	SHOC2	chr10:110964349-111011828	8	1909
145.	SLMAP	chr3:57757642-57927398	24	3263
146.	SNTA1	chr20:33408497-33443630	8	1678
147.	SOS1	chr2:38985814-39120432	23	4462
148.	SOS2	chr14:50118334-50231293	24	4626
149.	SPEG	chr2:219434968-219498199	50	11763
150.	SPRED1	chr15:38253176-38351674	7	1475
151.	SYNE1	chr6:152122426-152628341	154	30882
152.	SYNM	chr15:99105190-99133068	4	4778
153.	SYNPO2L	chr10:73646708-73655932	5	3134
154.	TAZ	chrX:154411834-154421014	10	1254
155.	TBX20	chr7:35202420-35253630	8	1508
156.	TBX5	chr12:114355522-114403908	8	1785
157.	TCAP	chr17:39665350-39666119	2	544
158.	TECRL	chr4:64277025-64409361	13	1371
159.	TGFB3	chr14:75959177-75980903	7	1383
160.	TMEM43	chr3:14125184-14141805	13	1652
161.	TMPO	chr12:98515858-98547868	10	3169
162.	TNNC1	chr3:52451265-52454025	6	606
163.	TNNI3	chr19:55151824-55157599	8	876
164.	TNNI3K	chr1:74235442-74543992	28	3237
165.	TNNT2	chr1:201359200-201373264	18	1307
166.	TPM1	chr15:63042820-63071182	17	1971
167.	TRDN	chr6:123218591-123636785	44	3418
168.	TRPM4	chr19:49157857-49211508	25	4149
169.	TTN	chr2:178527002-178804652	364	121875
170.	TTR	chr18:31591893-31598685	8	718

171.	VCL	chr10:73998198-74118179	22	3845
172.	ZIC3	chrX:137566682-137577281	4	1634

Supplementary Table S2

ARVC criteria of patient[1]

	Major criteria	Minor criteria	Dimension
Global or regional dysfunction and structural alterations	By 2D echo: ● Regional RV akinesia, dyskinesia, or aneurysm ● and 1 of the following (end diastole): — PLAX RVOT 32 mm (corrected for body size PLAX/BSA 19 mm/m^2) — PSAX RVOT 36 mm (corrected for body size PSAX/BSA 21 mm/m^2) — or fractional area change 33%<br>By MRI: ● Regional RV akinesia or dyskinesia or dyssynchronous RV contraction ● and 1 of the following: — Ratio of RV end-diastolic volume to BSA 110 mL/m^2 (male) or 100 mL/m^2 (female) — or RV ejection fraction 40%<br>By RV angiography: ● Regional RV akinesia, dyskinesia, or aneurysm	By 2D echo: ● Regional RV akinesia or dyskinesia ● and 1 of the following (end diastole): — PLAX RVOT 29 to 32 mm (corrected for body size PLAX/BSA 16 to 19 mm/m^2) — PSAX RVOT 32 to 36 mm (corrected for body size PSAX/BSA 18 to 21 mm/m^2) — or fractional area change from 33% to 40%<br>By MRI: ● Regional RV akinesia or dyskinesia or dyssynchronous RV contraction ● and 1 of the following: — Ratio of RV end-diastolic volume to BSA 100 to 110 mL/m^2 (male) or 90 to 100 mL/m^2 (female) — or RV ejection fraction from 40% to 45%	No criteria
Tissue characterization of wall	●Residual myocytes 60% by morphometric analysis (or 50% if estimated), with fibrous replacement of the RV free wall myocardium in 1 sample, with or without fatty replacement of tissue on endomyocardial biopsy	●Residual myocytes 60% to 75% by morphometric analysis (or 50% to 65% if estimated), with fibrous replacement of the RV free wall myocardium in 1 sample, with or without fatty replacement of tissue on endomyocardial biopsy	No criteria

Repolarization abnormalities	●Inverted T waves in right precordial leads (V_1, V_2, and V_3) or beyond in individuals more 14 years of age (in the absence of complete right bundle-branch block QRS 120 ms)	●Inverted T waves in leads V_1 and V_2 in individuals more 14 years of age (in the absence of complete right bundle-branch block) or in V_4, V_5, or V_6<br>●Inverted T waves in leads V_1, V_2, V_3, and V_4 in individuals more 14 years of age in the presence of complete right bundle-branch block	No criteria
Depolarization/conduction abnormalities	●Epsilon wave (reproducible low-amplitude signals between end of QRS complex to onset of the T wave) in the right precordial leads (V_1 to V_3)	● Late potentials by signal-averaged ECG in 1 of 3 parameters in the absence of a QRS duration of 110 ms on the standard ECG ● Filtered QRS duration (fQRS) 114 ms<br>● Duration of terminal QRS 40 µV (low-amplitude signal duration) 38 ms<br>● Root-mean-square voltage of terminal 40 ms 20 µV<br>● Terminal activation duration of QRS 55 ms measured from the nadir of the S wave to the end of the QRS, including R`, in V_1, V_2, or V_3, in the absence of complete right bundle-branch block	No criteria
Arrhythmias	● Nonsustained or sustained ventricular tachycardia of left bundle-branch morphology with superior axis (negative or indeterminate QRS in leads II, III, and aVF and positive in lead aVL)	● Nonsustained or sustained ventricular tachycardia of RV outflow configuration, left bundle-branch block morphology with inferior axis (positive QRS in leads II, III, and aVF and negative in lead aVL) or of unknown axis ●More 500 ventricular extrasystoles per 24 hours (Holter)	Minor criteria
Family history	● ARVC/D confirmed in a first-degree relative who meets current Task	● History of ARVC/D in a first-degree relative in whom it is not possible or practical	No criteria

	Force criteria<br>● ARVC/D confirmed pathologically at autopsy or surgery in a first-degree relative<br>● Identification of a pathogenic mutation categorized as associated or probably associated with ARVC/D in the patient under evaluation	to determine whether the family member meets current Task Force criteria<br>● Premature sudden death (35 years of age) due to suspected ARVC/D in a first-degree relative<br>● ARVC/D confirmed pathologically or by current Task Force Criteria in second-degree relative	

PLAX indicates parasternal long-axis view; RVOT, RV outflow tract; BSA, body surface area; PSAX, parasternal short-axis view; aVF, augmented voltage unipolar left foot lead; and aVL, augmented voltage unipolar left arm lead.

Diagnostic terminology for revised criteria: definite diagnosis: 2 major or 1 major and 2 minor criteria or 4 minor from different categories; borderline: 1 major and 1 minor or 3 minor criteria from different categories; possible: 1 major or 2 minor criteria from different categories.

Patient has only one minor criteria, this is not enough even for possible diagnosis.

1. Marcus FI, McKenna WJ et al. Diagnosis of arrhythmogenic right ventricular cardiomyopathy/dysplasia: proposed modification of the Task Force Criteria. Eur Heart J. 2010 Apr;31(7):806-14.
